# Occurrence of IgG antibodies against *Toxoplasma gondii*, *Neospora caninum*, and *Leptospira* spp. in goats and sheep from an indigenous village in Pernambuco, Brazil

**DOI:** 10.1590/S1984-29612023022

**Published:** 2023-04-28

**Authors:** Cynthia Maria Morais de Queiroz Galvão, Pollyanne Raysa Fernandes de Oliveira, André Luiz de Aguiar Cavalcanti, Denise Batista Nogueira, Sérgio Santos de Azevedo, Rafael Antônio do Nascimento Ramos, Rinaldo Aparecido Mota

**Affiliations:** 1 Departamento de Medicina Veterinária, Universidade Federal Rural de Pernambuco - UFRPE, Recife, PE, Brasil; 2 Programa de Pós-graduação em Epidemiologia Experimental Aplicada às Zoonoses, Universidade de São Paulo - USP, São Paulo, SP, Brasil; 3 Departamento de Medicina Veterinária, Universidade Federal Rural de Campina Grande - UFCG, Patos, PB, Brasil; 4 Departamento de Medicina Veterinária, Universidade Federal do Agreste de Pernambuco - UFAPE, Garanhuns, PE, Brasil

**Keywords:** Indigenous community, MAT, IFAT, zoonosis, serology test, Comunidade indígena, MAT, IFAT, zoonose, teste sorológico

## Abstract

This study aimed to determine the occurrence of anti-*Toxoplasma gondii*, *Neospora caninum*, and *Leptospira* spp. antibodies in sheep and goats raised in villages of the Xukuru do Ororubá indigenous community, Pernambuco, Brazil. A total of 180 serum samples from sheep and 108 serum samples from goats of both sexes and different ages were analyzed. For antibody research, indirect immunofluorescence antibody test (IFAT) were used for the protozoa *T. gondii* and *N. caninum*, and microscopic agglutination test (MAT) for *Leptospira* spp., with a cutoff titer of 1:64, 1:50 and 1:100, respectively. The frequency of anti-*T. gondii* antibodies was 16.6% (30/180) for sheep and 11.1% (12/108) for goats. The frequency of anti-*N. caninum* antibodies was 10.55% (19/180) for sheep, and 20.37% (22/108) for goats, while for *Leptospira* spp., 2.2% (4/180) of sheep and 1.85% (2/108) of goats reacted positively. The results obtained in this study are unprecedented in indigenous communities in the country and serve as an alert for monitoring goats and sheep from the Xukuru do Ororubá indigenous village regarding the occurrence and productive impact of infections by *T. gondii*, *N. caninum,* and *Leptospira* spp., in addition to the occurrence of the zoonosis toxoplasmosis and leptospirosis in the indigenous community.

## Resumo

Objetivou-se determinar a ocorrência de anticorpos anti-*Toxoplasma gon*dii, *Neospora caninum* e *Leptospira* spp., em ovinos e caprinos criados em aldeias da comunidade indígena Xukuru do Ororubá, Pernambuco, Brasil. Foram analisadas 180 amostras de soro de ovinos e 108 amostras de soro de caprinos de ambos os sexos e diferentes idades. Para a pesquisa de anticorpos foi utilizada a técnica de Reação de Imunofluorescência indireta (RIFI), para os protozoários *T.* gondii e *N. caninum* e Aglutinação Microscópica (MAT) para *Leptospira* spp., com ponto de corte de 1:64, 1:50 e 1:100, respectivamente. A frequência de anticorpos anti-*T gondii* foi de 16,6% (30/180) em ovinos e 11,1% (12/108) em caprinos. A frequência de anticorpos anti-*N. caninum* foi de 10,55% (19/180) para ovinos e 20,37% (22/108) para caprinos, enquanto para *Leptospira* spp., 2,2% (4/180) dos ovinos e 1,85% (2/108) dos caprinos reagiram positivamente. Os resultados obtidos neste estudo são inéditos em comunidades indígenas do país e alertam para o monitoramento de caprinos e ovinos da aldeia indígena Xukuru do Ororubá, quanto à ocorrência e impacto produtivo de infecções por *T. gondii*, *N. caninum* e *Leptospira* spp., além da ocorrência de zoonoses como a toxoplasmose e leptospirose na comunidade indígena.

The indigenous village of the Xukuru do Ororubá is situated in the mesoregion of the Agreste, state of Pernambuco, approximately 215 km from Recife (latitude 8º 05 South and longitude 34º 88 west of Greenwich), in the Serra do Ororubá, an officially recognized territory of the ethnic group (Silva, 2010). This territory has agricultural characteristics, considering the existence of water and a variable climate, ranging from humid areas to arid and rain-dependent regions (Silva, 2017). In addition to crops, the Xukuru do Ororubá raise cattle, horses, sheep, and goats, essentially in subsistence systems, and the producer surplus is sold at fairs (Oliveira, 2011; Silva, 2011).

Indigenous populations have encouraged agricultural activities since the establishment of the colonization process. In this process, animal husbandry forms an important colonizing model of land occupation, civilization of territories, and social groups (Silva, 2010). To date, the incentives for implementing animal husbandry on a larger scale in indigenous villages remain embedded in a civilizational bias (Velden, 2011). In the state of Pernambuco, ten indigenous individuals have been characterized in several ethnographic studies (IBGE, 2009); however, there is a considerable gap in the production/productivity and sanitary conditions of animals raised by these people.

Productivity in small ruminant farms is greatly influenced by reproductive efficiency, and among the multiple etiologies that may cause reproductive failure, infections by *Toxoplasma gondii*, *Neospora caninum* (apicomplexan protozoa) (Dubey, 2003, 2010), in addition to *Leptospira* spp. (Gram-negative bacteria) stand out (Ellis, 2015).

Toxoplasmosis is an anthropozoonosis caused by the protozoan *Toxoplasma gondii*. It has a cosmopolitan distribution and affects all homeothermic animals, causing asymptomatic infection to severe systemic conditions (Stelzer et al., 2019). Consumption of products derived from infected goats and sheep, such as raw milk or undercooked meat, represents a notable source of infection for humans (Dubey, 2010).

Neosporosis is a disease caused by *N. caninum*, recognized worldwide as a significant cause of abortions among cattle herds (Dubey, 2003). However, it has also been reported to cause reproductive failure in sheep and goats (Dubey, 2003, 2010).

Leptospirosis is a bacterial anthropozoonosis of worldwide distribution, causing epidemic outbreaks in animals and humans. It is transmitted by direct or indirect contact with the urine of infected animals (Ellis, 2015). It leads to economic damage in sheep and goats’ production due to infection, resulting in decreased fertility, neonatal deaths, and abortions (Ellis, 2015). In addition, these animals are potential sources of infection to other animals and humans, given their renal colonization by bacteria (Dubey, 2003, 2010; Suepaul et al., 2011).

Several studies have reported infection by *T. gondii*, *N. caninum,* and *Leptospira* spp. in sheep and goats in Brazil in different production systems (Dubey, 2003, 2010; Topazio et al., 2015); however, little is known about infection in animals from indigenous communities in Brazil. The elucidation of epidemiological aspects favors understanding the dynamics of diseases that compromise productivity within villages, as well as scales the risk of transmission of zoonotic agents by consumption of products obtained in this system (Jones et al., 2017).

The objective of this study was to determine the prevalence of antibodies anti-*T*. *gondii*, *N. caninum*, and *Leptospira* spp. in sheep and goats raised in villages of the Xukuru do Ororubá indigenous community, Pernambuco, Brazil.

Owing to the lack of information on the populations of sheep and goats in the villages of the Xukuru do Ororubá territory, a non-probabilistic sampling method was chosen. The target population consisted of animals of all ages, sexes, and breeds. Serological samples were collected from 180 sheep and 108 goats from Canabrava, Capim de Planta, Mascarenhas, Cimbres, and Caípe villages.

Blood samples (5mL) from the animals were obtained by venipuncture of the external jugular vein using vacuum tubes without anticoagulant. The samples were centrifuged at 1000×*g* for five minutes to obtain serum. Blood sera were aliquoted and stored at -20°C until serological tests were performed.

Crude antigens consisting of tachyzoites of *T*. *gondii* (ME-49 strain) and *N*. *caninum* (Nc-Spain7 strain) were used and maintained in monolayer culture of MA-104 (ATCC® CRL-2378.1).

Indirect immunofluorescence antibody test (IFAT) was used for the detection of anti-*T*. *gondii* (cutoff 1:64) and anti-*N*. *caninum* (cutoff 1:50) antibodies (Silva et al., 2012; Porto et al., 2016). Tachyzoites of *T. gondii* ME-49 strain and *N. caninum* Nc-Sp7 strain were used as antigens to sensitize the slides. Positive and negative control sera were included in all slides.

The microscopic agglutination test (MAT) was performed using a collection of live antigens cultured in Ellinghausen-McCullough-Johnson-Harris medium, free from contamination and autoagglutination, represented by the following serovars: *Leptospira* interrogans (serovars: Bratislava, Canicola, Djasiman, Australis, Hardjoprajitno, Icterohaemorrhagiae, Pomona, Bataviae), *Leptospira* borgpetersenii (serovars: Castellonis, Javanica, Hardjobovis, and Tarassovi), *Leptospira* kirschneri (serovar: Grippotyphosa), *Leptospira* santarosai (serovar: Shermani), *Leptospira* biflexa (serovar: Patoc).

Positive and negative control serum samples were used for the reactions. Initially, sera were screened at a 1:50 dilution and then subjected to titration by serial dilution. Samples with titers ≥50 were considered positive (Nogueira et al., 2020).

Data were tabulated, grouped, and analyzed by simple descriptive statistics, using absolute and relative frequency in the 2013 Microsoft Excel software.

IgG antibodies against *Toxoplasma gondii*, *Neospora caninum,* and *Leptospira* spp. in small ruminants raised in the villages of the Xukuru do Ororubá territory, in Pernambuco, are shown in Table 1.

**Table 1:** Serology results for *Toxoplasma gondii* and *Neospora caninum* and *Leptospira* spp. in sheep and goat samples from the Xukuru do Ororubá territory, Pernambuco.

Species	IFAT*				MAT**	
	*T. gondii*		*N. caninum*		*Leptospira* spp.	
	Positive	Negative	Positive	Negative	Positive	Negative
Sheep	16.6% (30/180)	83.3% (150/180)	10.55% (19/180)	89.4% (161/180)	2.2% (4/180)	97.7% (176/180)
Goat	11.1% (12/108)	88.8% (96/108)	20.37% (22/108)	79.63% (86/108)	1.85% (2/108)	98.14% (106/108)

* MAT: microscopic agglutination test;

** IFAT: indirect immunofluorescence antibody test.

It is noteworthy that 16.6% of sheep and 11.1% of goats were positive for *T. gondii* antibodies; 10.5% of sheep and 20.73% of goats were positive for *N. caninum*; 2.2% of sheep and 1.85% of goats were positive for *Leptospira* spp. The serovars detected and their respective titers were Bataviae (1/6; 16.6%; 50), Djasiman (1/6; 16.6%; 50), Pomona (1/6; 16.6%; 50), Shermani (1/6; 16.6%; 50), Patoc (2/6; 33.3%; 100), Australis (1/6; 16.6%; 50), and Tarassovi (1/6; 16.6%; 50). Some animals simultaneously reacted to more than one serovar.

The results obtained for *T. gondii* and *N. caninum* was within the positivity average of other studies carried out in the country (Silva et al., 2012; Romanelli et al., 2020; Costa et al., 2021). In addition to its importance for public health, *T.* gondii is one of the main abortifacient agents in small ruminants, and human infection can result in abortion or neurological infection in immunosuppressed individuals (Stelzer et al., 2019). Previous studies have reported the risk factors associated with the infection of indigenous people by *T. gondii*, highlighting the consumption of water from rivers, streams, and wells and the presence of felids near these sources of consumption (Santos et al., 2019), because of the dispersion of oocysts in the environment. In addition, transmission through the consumption of infected animal tissues is an important route of transmission of the protozoan (Dubey, 2010), which is an imminent risk to subsistence livestock. While collecting samples, we observed many domestic animals circulating in villages, such as dogs and cats. In addition, cats are widely used for rodent control in goat and sheep farms in the Xukuru territory. This habit can impact the frequency of seropositive sheep and goats since this and other wild felids are definitive hosts of *T. gondii* (Stelzer et al., 2019).

In Brazil, indigenous communities maintain a consumption model based on the practice of hunting, fishing, and extractivism with little external intervention; however, others have developed agricultural production models based on a subsistence model (Silva, 2011). In the territory of Xukuru do Ororubá, the animal production system presents precarious technical development, which favors the occurrence of diseases due to poor sanitary conditions. Goat and sheep productivity depends directly on their reproductive efficiency. Thus, abortion and neonatal mortality constitute one of the leading causes of economic losses within the productive sector or in subsistence creations (Stelzer et al., 2019).

In this indigenous community, the presence of dogs was also observed on goat and sheep properties. Domestic dogs are definitive hosts of *N. caninum* and eliminate oocysts that contaminate the environment, water, and pastures, favoring infection by protozoans (Dubey, 2003). Although *N*. *caninum* infection is established more efficiently in cattle, it deserves greater attention in the diagnosis of reproductive failure in sheep and goats (Porto et al., 2016; Oliveira et al., 2020), as abortion and stillbirths have been reported (Dubey, 2003). Serological and molecular studies have shown infection frequencies by *N. caninum* similar to or higher than those of *Toxoplasma gondii* in small ruminants (Dubey, 2003, 2010).

In the present study, the occurrence of animals positive for *Leptospira* spp. was relatively low (2.2% for sheep and 1.85% for goats). Serology for *Leptospira* spp. identified seven serovars (Patoc, Djasiman, Australis, Tarassovi, Shermani, Pomona, and Bataviae) with titers of 100. The goats and sheep of this indigenous community are not vaccinated against leptospirosis, so the antibodies detected refer to natural infection by this bacterium (Ellis, 2015). The most prevalent serovars in goats and sheep are Pomona, Hardjo Icterohaemorrhagiae, and Canicola (Cortizo et al., 2015; Topazio et al., 2015). However, the frequency of serovars detected in each animal species is significantly related to the reservoirs involved in the transmission chain in each region studied (Ellis, 2015), therefore, serovars may vary between areas. In Northeast of Brazil (Ceará, Paraíba, Piauí, Rio Grande do Norte, and Sergipe), the serovar Autumnalis is the most frequent in sheep (Silva et al., 2021). Additionally, worldwide investigations have detected serovars Pomona, Australis, and Tarassovi in goat species, while Australis, Tarassovi, Bratislava Grippothyphosa, and Hebdomadis were detected in sheep species (Guzman-Barragan et al., 2022; Hajikolaei et al., 2022). When the disease manifests in goats and sheep, it is characterized by reproductive disorders, such as infertility, abortion, neonatal death, and decreased milk production (Cortizo et al., 2015; Campos et al., 2017).

In this study, it was not possible to determine the impact of these infections in goats and sheep in terms of reproductive disorders, or the relationship of these animals as sources of infection for native people. However, considering the poor sanitary conditions observed in the village and the habit of consuming meat and animal viscera, it is prudent to consider the occurrence of toxoplasmosis and leptospirosis among indigenous people. In this way, the results of this study will be able to guide measures to prevent these diseases in humans and animals, thus contributing to the maintenance of the health and quality of life of native people in Brazil.

The results obtained in this study are unprecedented in indigenous communities and serve as an alert for monitoring the village's goats and sheep. Xukuru do Ororubá indigenous people regarding the occurrence and productive impact of infections with *T. gondii*, *N. caninum*, and *Leptospira* spp., in addition to the occurrence of toxoplasmosis and leptospirosis in the indigenous community.
